# Contributions to the study of the relationship between blood pressure and mental health

**DOI:** 10.1192/j.eurpsy.2021.1255

**Published:** 2021-08-13

**Authors:** G. Esgalhado, A. Louro

**Affiliations:** Psychology And Education, University of Beira Interior, Covilha, Portugal

**Keywords:** Cardiovascular indicators, Blood pressure, mental health, Psychopathological symptomatology

## Abstract

**Introduction:**

Blood pressure (BP) refers to the pressure that the blood exerts on the walls of blood vessels. There is a number of evidences that show that depression, anxiety, and also stress have a high incidence in people suffering from hypertension.

**Objectives:**

To correlate blood pressure levels with psychopathological symptomatology levels and to compare differences between genders, age and education levels.

**Methods:**

This was a descriptive, inferential and correlational study encompassing 1407 participants, aged from 18 – 89 years of age (average age = 36 years). Measures included biomedical data - systolic blood pressure and diastolic blood pressure, arrhythmias and heartbeat, as well as the demographic variables - sex, age and education of the participants; mental health (psychopathological symptomatology) was measured using the Portuguese version of the BSI.

**Results:**

In the present study there were no statistically significant relationships between Systolic Blood Pressure, Diastolic Blood Pressure and General Symptom Index, as well as for each BSI dimension. Nevertheless, statistically significant differences were found between Diastolic Blood Pressure and obsessions-compulsions, interpersonal sensibility and hostility. Also, there are statistically significant differences for BP according to gender and education.

**Conclusions:**

This study brings important contributions to the study of the associations between blood pressure and mental health, with import implications for intervention and prevention.

